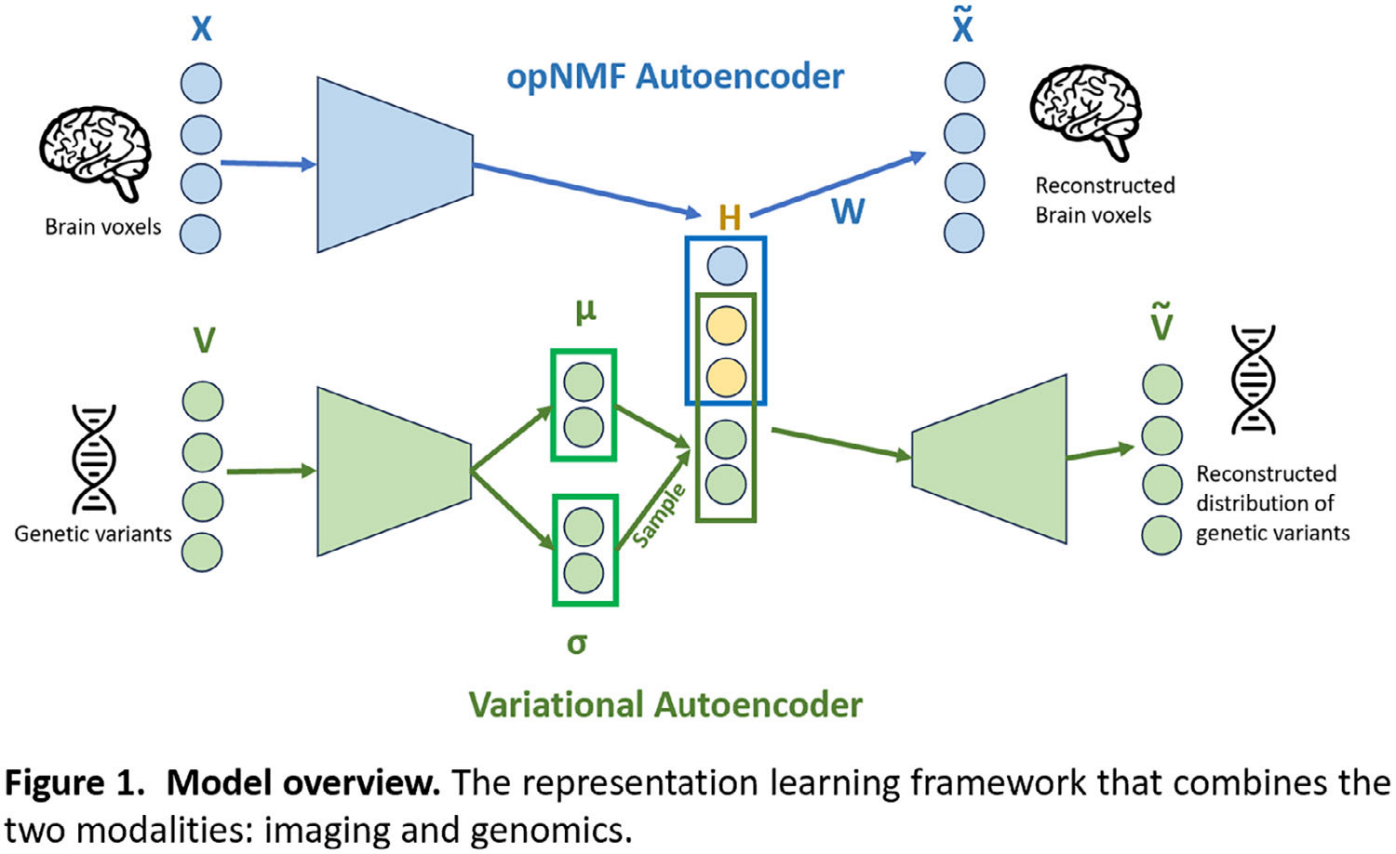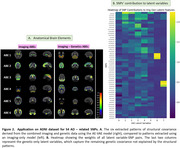# Genetically‐informed parcellation reveals interpretable brain patterns and enhanced genetic associations in Alzheimer's Disease compared to structural‐only approaches

**DOI:** 10.1002/alz70856_098832

**Published:** 2025-12-24

**Authors:** Marilena De Pian, Christos Davatzikos

**Affiliations:** ^1^ University of Pennsylvania, Philadelphia, PA, USA; ^2^ Artificial Intelligence in Biomedical Imaging Laboratory (AIBIL), Center for and Data Science for Integrated Diagnostics (AI2D), Perelman School of Medicine, University of Pennsylvania, Philadelphia, PA, USA

## Abstract

**Background:**

Advances in brain imaging genetics have propelled precision medicine by generating imaging‐derived phenotypes linked to genetic variants, particularly in polygenic neurodegenerative diseases like Alzheimer's Disease (AD), where genetic variants exert pleiotropic effects across the genome. Using a representation learning framework, we provide interpretable summaries of brain structural networks guided by brain MRI and genetic variations.

**Method:**

We applied a combination of two generative models—Autoencoder and Variational Autoencoder—to identify genetically influenced brain structural patterns from MRI data. This approach partitions the brain into anatomical brain elements (ABEs) that correlate with genetic variations, including disease‐related ones, in a data‐driven manner (Figure 1). The model was applied to clinical data from healthy controls, AD, and Mild Cognitive Impairment (MCI) patients (*N* = 1564) from the Alzheimer's Disease Neuroimaging Initiative (ADNI) dataset. Using voxel‐wise brain volumetric measurements and 54 AD‐related Single Nucleotide Polymorphisms (SNPs), we extracted genetic‐correlated ABEs.

**Result:**

The model robustly detected specific imaging and genetic patterns and elucidated their associations. When applied to ADNI data, it grouped brain regions based on structural and genetic covariance, creating a novel brain parcellation distinct from those based solely on structural covariance. Six genetically informed ABEs were extracted (Figure 2A). Notably, the subcortical regions and orbitofrontal cortex were clustered together, distinct from the rest of the frontal lobe. Most cerebellar regions were grouped together, except the inferior posterior lobe of the cerebellar vermis and hemisphere. These distinctions were absent when genetic information was excluded. We also pinpointed specific SNPs that were associated with each ABE. For instance, rs429358 (APOE) and rs41289512 (NECTIN2) were associated with temporal and hippocampal ABEs, aligning with existing literature and several ABCA7 polymorphisms (rs4147929, rs3752246, rs111278892) and CASS4 polymorphisms (rs7274581, rs6014724, rs6024870) were clustered together in association with specific ABEs respectively (Figure 2B).

**Conclusion:**

These insights highlight a novel brain atlas that captures the complexity of genetic and neuroanatomical heterogeneity, refining our understanding of how genetic factors influence brain anatomy.